# What is the effect of a decision aid in potentially vulnerable parents? Insights from the head CT choice randomized trial

**DOI:** 10.1111/hex.12965

**Published:** 2019-11-23

**Authors:** Rachel M. Skains, Nathan Kuppermann, James L. Homme, Anupam B. Kharbanda, Leah Tzimenatos, Jeffrey P. Louie, Daniel M. Cohen, Lise E. Nigrovic, Jessica J. Westphal, Nilay D. Shah, Jonathan Inselman, Michael J. Ferrara, Jeph Herrin, Victor M. Montori, Erik P. Hess

**Affiliations:** ^1^ Department of Emergency Medicine University of Alabama at Birmingham Birmingham AL USA; ^2^ Departments of Emergency Medicine and Pediatrics University of California Davis School of Medicine University of California Davis Health Sacramento CA USA; ^3^ Division of Pediatric Emergency Medicine Departments of Emergency Medicine and Pediatrics Mayo Clinic Rochester MN USA; ^4^ Department of Pediatric Emergency Medicine Children’s Hospitals and Clinics of Minnesota Minneapolis MN USA; ^5^ Department of Emergency Medicine University of California Davis School of Medicine University of California Davis Health Sacramento CA USA; ^6^ Division of Pediatric Emergency Medicine Department of Pediatrics University of Minnesota Minneapolis MN USA; ^7^ Division of Emergency Medicine Nationwide Children’s Hospital Columbus OH USA; ^8^ Division of Emergency Medicine Boston Children’s Hospital Boston MA USA; ^9^ Parent Representative Rochester MN USA; ^10^ Division of Health Care Policy and Research Department of Health Sciences Research Mayo Clinic College of Medicine Rochester MN USA; ^11^ Mayo Clinic Robert D. and Patricia E. Kern Center for the Science of Healthcare Delivery Rochester MN USA; ^12^ Division of Trauma, Critical Care and General Surgery Departments of Emergency Medicine and Surgery Mayo Clinic College of Medicine Rochester MN USA; ^13^ Yale University School of Medicine New Haven CT USA; ^14^ Health Research & Educational Trust Chicago IL USA; ^15^ Knowledge and Evaluation Research Unit Department of Internal Medicine Mayo Clinic Rochester MN USA; ^16^ Department of Emergency Medicine Mayo Clinic Rochester MN USA

**Keywords:** decision aid, head trauma, paediatrics, shared decision making

## Abstract

**Objective:**

To test the hypotheses that use of the *Head CT Choice* decision aid would be similarly effective in all parent/patient dyads but parents with high (vs low) numeracy experience a greater increase in knowledge while those with low (vs high) health literacy experience a greater increase in trust.

**Methods:**

This was a secondary analysis of a cluster randomized trial conducted at seven sites. One hundred seventy‐two clinicians caring for 971 children at intermediate risk for clinically important traumatic brain injuries were randomized to shared decision making facilitated by the DA (n = 493) or to usual care (n = 478). We assessed for subgroup effects based on patient and parent characteristics, including socioeconomic status (health literacy, numeracy and income). We tested for interactions using regression models with indicators for arm assignment and study site.

**Results:**

The decision aid did not increase knowledge more in parents with high numeracy (*P* for interaction [*P*_int_] = 0.14) or physician trust more in parents with low health literacy (*P*_int_ = 0.34). The decision aid decreased decisional conflict more in non‐white parents (decisional conflict scale, −8.14, 95% CI: −12.33 to −3.95; *P*_int_ = 0.05) and increased physician trust more in socioeconomically disadvantaged parents (trust in physician scale, OR: 8.59, 95% CI: 2.35‐14.83; *P*_int_ = 0.04).

**Conclusions:**

Use of the *Head CT Choice* decision aid resulted in less decisional conflict in non‐white parents and greater physician trust in socioeconomically disadvantaged parents. Decision aids may be particularly effective in potentially vulnerable parents.

## 1 INTRODUCTION

Shared decision making (SDM) aims to improve health‐care quality by involving patients, parents and clinicians in medical decisions. Decision aids (DAs), patient‐centred tools that facilitate SDM, have been shown to improve patients’ knowledge, decrease decisional conflict and enhance patient engagement in decision making.^1^, ^2^, ^3^, ^4^, ^5^ Although SDM is an emerging trend in paediatrics, few interventions to promote SDM in paediatric emergency care have been rigorously studied.^5^ Further, there is limited research on the impact of SDM in various population subgroups such as those with low health literacy or numeracy or among individuals of different ethnic/racial groups.

**Figure 1:**
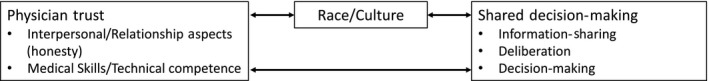
Conceptual model of the relationships between shared decision making, race/culture and physician trust. In this model, the shared decision‐making domains of information sharing, deliberation and decision making influence physician trust in addition to race and culture. (Reproduced with permission from *Health Communications*.)

Recently, a multicentre randomized trial evaluated the impact of a DA, Chest Pain Choice, in patients with chest pain at low risk for acute coronary syndrome. In a planned secondary analysis, the DA aided all subgroups to a similar extent, with greater knowledge transfer in patients with high numeracy and greater physician trust in patients with low health literacy.^6^ A qualitative study of African American patients with diabetes further explores the relationship between shared decision making and patient trust.^7^ In focus groups and in‐depth interviews, participants revealed concerns about potential racial bias and whether their physician might withhold medical information from them. At the same time, patients described specific physician shared decision‐making behaviours, such as information sharing and discussing the pros and cons of treatment options, as enhancers of patient trust. Guided by these insights, the authors proposed a conceptual model in which both shared decision‐making behaviours and race independently influence physician trust (Figure 1). In this model, the information sharing, deliberation and decision‐making domains of shared decision making have potential to enhance patient trust, even in the context of racial and cultural differences between patients and their physicians.

A recently completed multicentre randomized trial evaluating the impact of a DA in parents of children with minor head trauma, ‘*Head CT Choice*’, offers a unique opportunity to evaluate potential subgroup effects of a DA in the context of paediatric emergency care. In this trial, 172 clinicians caring for 971 children at intermediate risk for clinically important traumatic brain injuries (ciTBIs) were cluster‐randomized to SDM facilitated by a DA or to usual care. Similar to what has been observed in adult SDM trials,^1^, ^2^, ^3^ parents randomized to shared decision making had greater knowledge, less decisional conflict and were more engaged in decision making compared to usual care.^8^

Based on the summary effect estimates of DAs published in two meta‐analyses^2^, ^5^ an insightful qualitative study exploring the relationship between physician trust and shared decision making,^7^ and a recent subgroup analysis of the effects of a DA in adults with chest pain,^6^ we hypothesized that use of the *Head CT Choice* DA would be similarly effective in all parent/patient dyads but would increase knowledge more in parents with high numeracy and increase physician trust more in parents with low health literacy.

## 2 DESIGN AND METHODS

### 2.1 Study design

This was a planned secondary analysis of a SDM trial in parents of children with minor head trauma. The study protocol has been previously published.^9^ The trial was conducted at seven clinical sites across the United States, including an academic emergency department (ED) serving a largely rural population (Mayo Clinic, Rochester, MN), four academic EDs serving urban populations (University of California Davis Medical Center, Sacramento CA; University of Minnesota Masonic Children's Hospital, Minneapolis, MN; Nationwide Children's Hospital, Columbus, Ohio; and Boston Children's Hospital, Boston, Massachusetts) and two community paediatric EDs serving urban/suburban populations (Children's Hospitals and Clinics of Minnesota EDs in Minneapolis and Saint Paul, Minnesota, respectively). Emergency clinicians caring for patients at intermediate risk for ciTBI were randomized to use the DA or to usual care. Approval to conduct the trial was obtained from the Institutional Review Board at each participating site. Written informed consent was obtained from each participating clinician and parent, and assent was obtained from children 12 years or older prior to enrolment.

### 2.2 Participants

Eligible patients included children (<18 years old) seen in the ED for minor blunt head trauma, defined as a Glasgow Coma Scale (GCS) score of 15 after non‐negligible traumatic mechanisms (ie, excluding ground‐level falls and running into stationary objects) within 24 hours of injury. Eligible patients had 1 or 2 Pediatric Emergency Care Applied Research Network (PECARN) intermediate risk factors for ciTBI.^10^

### 2.3 Study treatments

#### 2.3.1 Intervention clinicians

The *Head CT Choice* DA was developed in Rochester, MN, USA, through a participatory action research methodology that involved eliciting input from a multidisciplinary investigative team including clinicians, health services researchers, a graphic designer, a radiation physicist and parent stakeholders. An initial DA prototype was designed based on input from the investigative team and subsequently refined based on feedback received from parents and clinicians after use in clinical encounters. Full details of the DA development process have been described previously.^9^ The DA can be accessed at https://shareddecisions.mayoclinic.org/decision-aid-information/head-ct-choice-decision-aid/.

After enrolment, study coordinators calculated the patient's precise PECARN risk estimate of ciTBI (calculated based on the presence or absence of individual PECARN clinical predictors in isolation, as well as combinations of predictors^10^) and provided intervention clinicians a DA corresponding to the individual patient's level of risk. Research staff offered intervention clinicians a brief, just‐in‐time refresher of DA content and use just prior to the clinical encounter. Clinicians then brought the DA to the bedside and used it during the clinical encounter to facilitate a SDM discussion with the parents.

#### 2.3.2 Usual care clinicians

For patients whose clinicians were randomized to usual care, research assistants instructed the clinicians to discuss management options with parents according to each clinician's usual fashion. Clinicians in the usual care arm were blinded to the precise risk estimates for ciTBI calculated from the PECARN head injury public access database and did not have access to the DA. The usual care arm was not standardized.

### 2.4 Data collection

Data documenting the process of screening and enrolment were collected in compliance with CONSORT guidelines.^11^ Patient characteristics collected included the sex and age of the child as well as the number and type of PECARN risk factors for ciTBI. Parent characteristics collected included their race, highest level of education, health literacy, numeracy and annual household income. We also recorded the number of parents present during the encounter (father only, mother only and/or both parents). Outcome data analysed in this report were obtained from video and audio recordings of the parent‐clinician encounter, a pre‐encounter parent survey, a post‐encounter parent survey, a post‐encounter clinician survey, review of the electronic medical record and telephone follow‐up initiated 7 days after the ED visit. The parent and clinician surveys have been previously published.^9^ On the pre‐encounter survey, parent literacy was assessed using the subjective literacy scale^12^, ^13^ and numeracy was assessed using the subjective numeracy scale.^14^ The subjective literacy scale, which ranges from 3 to 15, consists of three items (each with a five‐point Likert response) that were summed to a total score after reverse coding one item, with higher scores indicating higher health literacy. The subjective numeracy scale, which quantifies an individual's ability to understand and use numbers in daily life, consists of an 8‐item survey. Responses to all 8 questions were reversed and averaged, creating an overall score ranging from 6 to 48, with higher scores indicating higher numeracy. The post‐encounter parent survey collected data assessing parents’ knowledge regarding their child's risk for ciTBI and the available management options.

### 2.5 Outcomes

The primary outcome, which was selected by parent stakeholders, was parent knowledge regarding their child's risk for ciTBI and the available diagnostic options. Parent knowledge was assessed by immediate post‐visit survey. Secondary outcomes, also obtained by post‐visit survey, included the degree of uncertainty parents experienced related to choosing between diagnostic options with which they were unfamiliar using the validated Decisional Conflict Scale (DCS)^15^ and parents’ trust in their clinician measured using the validated Trust in Physician Scale (TPS).^16^ Trained research assistants viewed encounter video recordings to assess the degree to which clinicians engaged parents in the decision‐making process using the 12‐item ‘observing patient involvement’ (OPTION) scale.^17^ To measure health‐care utilization, data were collected on the proportion of children who underwent cranial CT scanning during the ED visit, the most immediate utilization decision. Finally, the safety of DA use was assessed by comparing the rate of ciTBI in each arm of the trial.

### 2.6 Subgroups

We dichotomized parent and patient characteristics to assess the differential effect of the DA. The following patient characteristics were dichotomized: the sex and age in years (<2 years and 2‐18 years, as there are two different PECARN prediction rules based on this age cut‐off) and the number of PECARN risk factors (1 vs 2). The following parent characteristics were also dichotomized: race, highest level of education, health literacy, numeracy, annual household income and the number of parents present during the encounter. To explore the differential effectiveness of the DA in potentially vulnerable parents while simultaneously limiting the risk of bias associated with multiple testing, we created a combination variable to identify a socioeconomically disadvantaged parent subgroup. If the parent was of non‐white race, low health literacy or numeracy, and low income (<$40 000), they were classified as socioeconomically disadvantaged. We dichotomized the data for two reasons: (a) to avoid subgroups that were too small to analyse and (b) to simplify the analysis and interpretation of subgroup effects by way of interactions.^18^ Classifications for each variable were selected based on the distribution of the data, which we report in full, and conceptual considerations regarding the mostly likely contrasts to show heterogeneity of effect.

Classifications were as follows: sex of the child as ‘Male’ vs ‘Female’; race of the child as ‘White’ vs ‘Non‐White’; highest level of parent education as ‘Less than or equal to high school/ General Education Development (GED)’ vs ‘Greater than high school/ GED’; annual household income as ‘Less than $40 000’ vs ‘Greater than or equal to $40 000’; parent health literacy as ‘Typical (≥12)’ vs ‘Low (<12)’; parent numeracy as ‘Typical (≥34)’ vs ‘Low (<34)’; age of the child as ‘Less than 2 (<2)’ vs ‘Greater than or equal to 2 (≥2)’; number of PECARN risk factors as ‘1’ vs ‘2’; number of parents present during encounter as ‘1 Parent’ vs ‘2 Parents’; father only present during encounter as ‘No’ vs ‘Yes’; mother only present during encounter as ‘No’ vs ‘Yes’; and socioeconomically disadvantaged as ‘No’ vs ‘Yes’. Patients/parents missing a subgroup variable were excluded from the analysis for that subgroup. For race, the ‘Other’ category was included with ‘Non‐White’. For education, the ‘Other’ category was excluded from the dichotomous groups, as we did not assume that ‘other’ indicated either of the two categories. For the parent(s) present during the encounter, the ‘other’ category was excluded from the analysis for that subgroup (n = 8), where ‘other’ could indicate a family member, caregiver or friend.

### 2.7 Statistical analysis

Sample size and power estimates for the primary and secondary outcomes of the trial have been published elsewhere.^9^ Briefly, we estimated that enrolling 950 patients would provide 99% power to detect a 16% difference in parent knowledge between the DA and usual care arms. This percentage difference was selected a priori, as it had been observed in a prior pilot trial conducted in the ED setting.^3^ As with the primary analysis of the trial,^8^ outcome assessments for this analysis were measured using regression models (linear for continuous outcomes, multinomial for categorical outcomes) that included indicators for arm assignment and study site. To assess the effect of the DA in each subgroup, we included an interaction term for group assignment. To improve interpretation, we also replicated the primary trial analysis (ie, without an interaction term) within each subgroup and report whether the group effect differed significantly from zero. This group effect was reported as a coefficient for continuous outcomes and as odds ratios for dichotomous or multinomial outcomes.

The analytic approach was informed by publication guidelines for reporting subgroup analyses.^18^ Interaction testing between parent/patient characteristics and the outcomes of parent knowledge, decisional conflict, parent engagement in decision making and physician trust were pre‐specified, and a significance level of 5% was used to identify significant interactions for these subgroup effects. Significant interactions identified in subgroup analyses that were not pre‐specified were considered hypothesis generating. All analyses were performed using Stata 14.1 (2016. Stata Corporation).

The funder of the study had no role in study design, data collection, data analysis, data interpretation or writing of the report. All researchers’ maintained independence from the funder of the study.

## 3 RESULTS

From 1 April 2014 to 30 September 2016, we enrolled and randomized 172 clinicians (88 DA, 84 usual care) who later cared for 971 eligible children at intermediate risk of ciTBI (493 DA, 478 usual care). Data demonstrating the fidelity of screening and enrolment procedures and the completeness of follow‐up assessments are reported elsewhere.^9^

Table 1 shows parent and patient sociodemographic characteristics. The two parent/patient groups were similar. More than half of head‐injured children were white males. Approximately one‐half of the parents had less than a college degree. One‐third of parents reported low numeracy and one‐sixth low health literacy. Finally, in the majority of encounters, there was only one parent present.

**Table 1:** Baseline characteristics

Variable	Total (n = 971)	Usual Care (n = 478)	Decision Aid (n = 493)	*P* value
**Sex of child**				
Male	575 (59.1)	285 (59.6)	290 (58.8)	.8
Female	396 (40.8)	193 (40.4)	203 (41.2)	
**Parent race**				
White	718 (73.9)	347 (72.6)	371 (75.3)	.345
Non‐white	253 (26.1)	131 (27.4)	122 (24.7)	
**Parent education**				
Some high school or less	58 (6.0)	31 (6.5)	27 (5.5)	.261
High school or GED	101 (10.4)	55 (11.5)	46 (9.3)	
Some college/associates degree	278 (28.6)	126 (26.4)	152 (30.8)	
College graduate (4‐y)	298 (30.7)	147 (30.8)	151 (30.6)	
Graduate/professional	194 (20.0)	101 (21.1)	93 (18.9)	
Other	22 (2.3)	7 (1.5)	15 (3.0)	
Missing	20 (2.1)	11 (2.3)	9 (1.8)	
**Family annual income**				
Less than $20 000	129 (13.3)	66 (13.8)	63 (12.8)	.568
$20 000‐$29 999	56 (5.8)	26 (5.4)	30 (6.1)	
$30 000‐$39 999	79 (8.1)	34 (7.1)	45 (9.1)	
$40 000‐$59 999	115 (11.8)	55 (11.5)	60 (12.2)	
$60 000‐$79 999	95 (9.8)	51 (10.7)	44 (8.9)	
$80 000‐$99 999	107 (11.0)	60 (12.6)	47 ( 9.5)	
$100 000 or more	353 (36.4)	168 (35.1)	185 (37.5)	
Missing	37 (3.8)	18 (3.8)	19 (3.9)	
**Parent health literacy**				
Typical (≥12)	807 (83.1)	401 (83.9)	406 (82.4)	.48
Low (<12)	142 (14.6)	66 (13.8)	76 (15.4)	
Missing	22 (2.3)	11 (2.3)	11 (2.2)	
**Parent numeracy**				
Typical (≥34)	655 (67.5)	320 (66.9)	335 (68.0)	.738
Low (<34)	316 (32.5)	158 (33.1)	158 (32.0)	
**Adult present**				
Both parents	326 (33.6)	171 (35.8)	155 (31.4)	.357
One parent	637 (65.6)	303 (63.4)	334 (67.7)	
Other	8 (0.8)	4 (0.8)	4 (0.8)	
**Number of PECARN ciTBI risk factors**				
1	780 (80.3)	380 (79.5)	400 (81.1)	.521
2	191 (19.7)	98 (20.5)	93 (18.9)	

Note

Values are presented as number (%) unless otherwise indicated.

Abbreviations: ciTBI, clinically important traumatic brain injury; GED, graduate education diploma; PECARN, (Pediatric Emergency Care Applied Research Network); SD, standard deviation.

**Figure 2:**
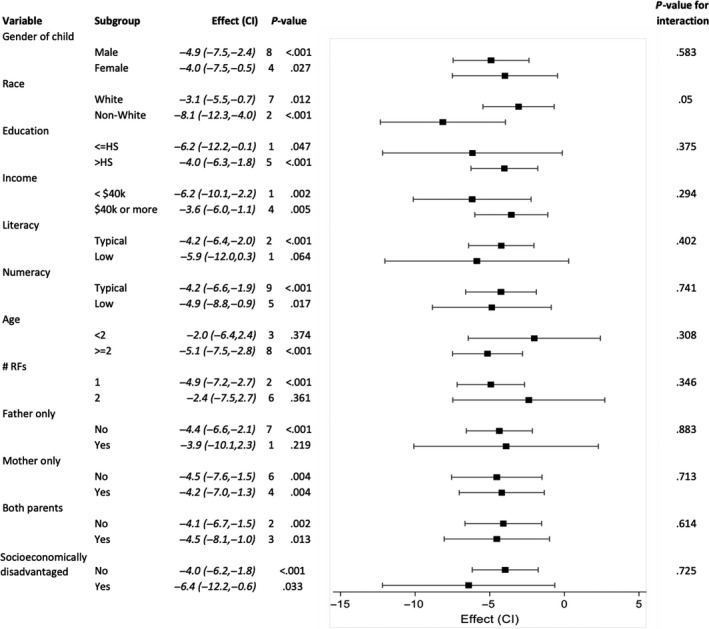
Decisional Conflict Scale subgroup effects. Forest plot demonstrating the effect of the *Head CT Choice* decision aid on parent decisional conflict in subgroups according to patient and parent characteristics

**Figure 3:**
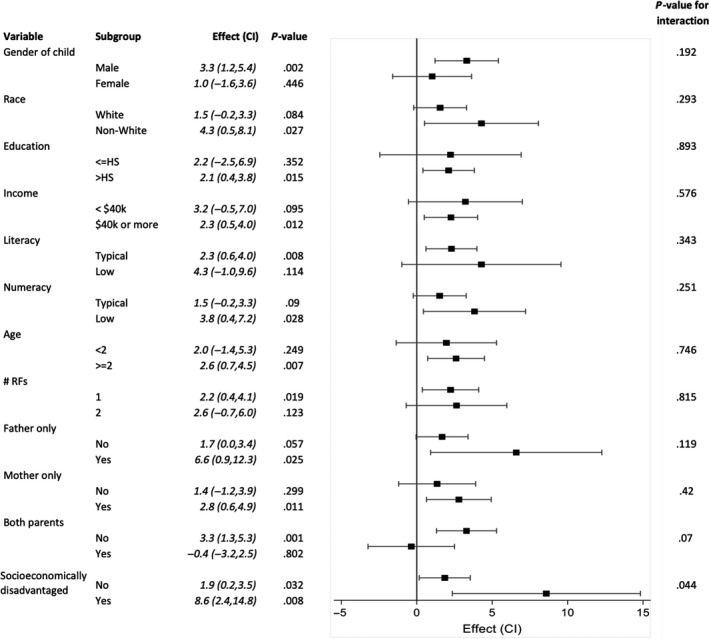
Trust in Physician Scale subgroup effects. Forest plot demonstrating the effect of the *Head CT Choice* decision aid on parent trust in the physician in subgroups according to patient and parent characteristics

In children with minor head trauma at intermediate risk of clinically important TBI according to the PECARN prediction rules (one or two intermediate risk PECARN factors),^10^ SDM facilitated by the *Head CT Choice* DA increased parent knowledge, decreased decisional conflict, increased parents’ trust in the clinician and facilitated greater clinician engagement of parents similarly across all patient and parent subgroups (Table 2). There was a significant interaction between patient race and the outcome of decisional conflict. The DA decreased decisional conflict to a relatively greater degree in non‐white parents compared to white parents (Table 2; Figure 2). There was also a significant interaction between whether the parent was socioeconomically disadvantaged and physician trust. The degree to which trust improved with use of the DA was greater in socioeconomically disadvantaged parents (Table 2; Figure 3). Interestingly, there was an inverse correlation between decisional conflict and physician trust (correlation = −0.40, *P* < .001). There were no significant interactions between parent/patient characteristics and the outcomes of knowledge or parent engagement in decision making (OPTION score).

**Table 2:** Differential effect of the decision aid on parent knowledge, decisional conflict, trust in the physician and involvement in the decision (OPTION Score) based on patient and parent sociodemographic characteristics

Characteristic	Knowledge (% questions correct)		Decisional Conflict		Trust in Physician		OPTION Score	
	Decision Aid Effect (OR, 95% CI)	*P* value	Decision Aid Effect (OR, 95% CI)	*P* value	Decision Aid Effect (OR, 95% CI)	*P* value	Decision Aid Effect (OR, 95% CI)	*P* value
Sex of child^a^								
Male	9.47 (6.30, 12.65)	.774	−4.91 (−7.46, −2.37)	.583	3.31 (1.21, 5.41)	.192	12.23 (10.55, 13.91)	.439
Female	8.88 (4.74, 13.03)		−3.98 (−7.50, −0.46)		1.02 (−1.60, 3.63)		11.08 (8.98, 13.18)	
Race								
White	9.70 (6.91, 12.48)	.199	−3.07 (−5.45, −0.68)^b^	.05	1.55 (−0.21, 3.30)	.293	12.27 (10.75, 13.79)	.17
Non‐white	5.91 (0.84, 10.97)		−8.14 (−12.33, −3.95)^b^		4.29 (0.51, 8.06)		9.98 (7.29, 12.67)	
Education								
≤HS	6.49 (0.55, 13.53)	.619	−6.16 (−12.18, −0.14)	.375	2.23 (−2.45, 6.92)	.893	12.55 (9.70,15.40)	.762
>HS	9.46 (6.85, 12.07)		−4.02 (−6.26, −1.78)		2.11 (0.41, 3.82)		11.81 (10.30,13.33)	
Income								
<$40k	9.53 (4.73, 14.33)	.999	−6.18 (−10.13, −2.23)	.294	3.23 (−0.55, 7.00)	.576	12.90 (10.50, 15.30)	.367
≥$40k	9.65 (6.85, 12.45)		−3.55 (−6.00, −1.11)		2.26 (0.49, 4.03)		11.35 (9.75, 12.95)	
Health literacy								
Typical	8.25 (5.60, 10.90)	.063	−4.23 (−6.42, −2.04)	.402	2.30 (0.61, 3.99)	.343	11.85 (10.40,13.31)	.802
Low	14.61 (7.36, 21.85)		−5.87 (−12.03, 0.29)		4.28 (−0.99, 9.56)		11.78 (8.57,14.99)	
Numeracy								
Typical	10.22 (7.36, 13.08)	.142	−4.25 (−6.62, −1.88)	.741	1.52 (−0.23, 3.28)	.251	11.51 (9.89, 13.13)	.756
Low	6.50 (1.89, 11.12)		−4.86 (−8.84, −0.88)		3.82 (0.44, 7.21)		11.72 (9.51, 13.93)	
Child age^a^								
<2	8.89 (3.49, 14.30)	.764	−2.02 (−6.45, 2.42)	.308	1.96 (−1.37, 5.29)	.746	12.18 (9.57, 14.79)	.681
≥2	9.42 (6.58, 12.27)		−5.15 (−7.49, −2.80)		2.61 (0.72, 4.49)		11.48 (9.95, 13.01)	
Number of PECARN ciTBI risk factors^a^								
1	8.88 (6.03, 11.73)	.662	−4.93 (−7.19, −2.67)	.346	2.24 (0.37, 4.11)	.815	11.34 (9.88,12.80)	.132
2	10.15 (4.82, 15.47)		−2.38 (−7.47, 2.71)		2.64 (−0.70, 5.97)		14.07 (10.96,17.18)	
Father only								
No	8.53 (5.89, 11.18)	.258	−4.36 (−6.57, −2.14)	.883	1.68 (−0.04, 3.40)	.119	11.51 (10.13,12.89)	.495
Yes	14.40 (5.43, 23.37)		−3.90 (−10.09, 2.28)		6.59 (0.92, 12.27)		12.75 (8.11,17.39)	
Mother only								
No	8.08 (4.24, 11.91)	.568	−4.53 (−7.56, −1.50)	.713	1.35 (−1.20,3.90)	.420	11.73 (9.68,13.78)	.820
Yes	10.11 (6.80, 13.43)		−4.20 (−7.05, −1.34)		2.79 (0.64,4.94)		11.68 (9.96,13.40)	
Both parents								
No	10.41 (7.33, 13.49)	.219	−4.09 (−6.66, −1.52)	.614	3.30 (1.31, 5.29)	.070	12.06 (10.45, 13.68)	.529
Yes	6.34 (2.06, 10.62)		−4.53 (−8.06, −1.00)		−0.37 (−3.23, 2.50)		11.36 (9.02, 13.70)	
Socioeconomically disadvantaged								
No	9.25 (6.63, 11.87)	.298	−3.96 (−6.17, −1.75)	.725	1.85 (0.16, 3.55)^b^	.044	11.86 (10.46, 13.27)	.569
Yes	6.65 (−1.10, 14.39)		−6.41 (−12.19, −0.64)		8.59 (−2.35, 14.83)^b^		10.57 (5.97, 15.16)	

Abbreviations: CI, confidence interval; OR, odds ratio.

a Indicates a patient characteristic.

b Indicates a significant decision aid effect for the subgroup compared with its control (usual care) for the outcome. Reported if the *P* value for the overall interaction is significant.

**Figure 4:**
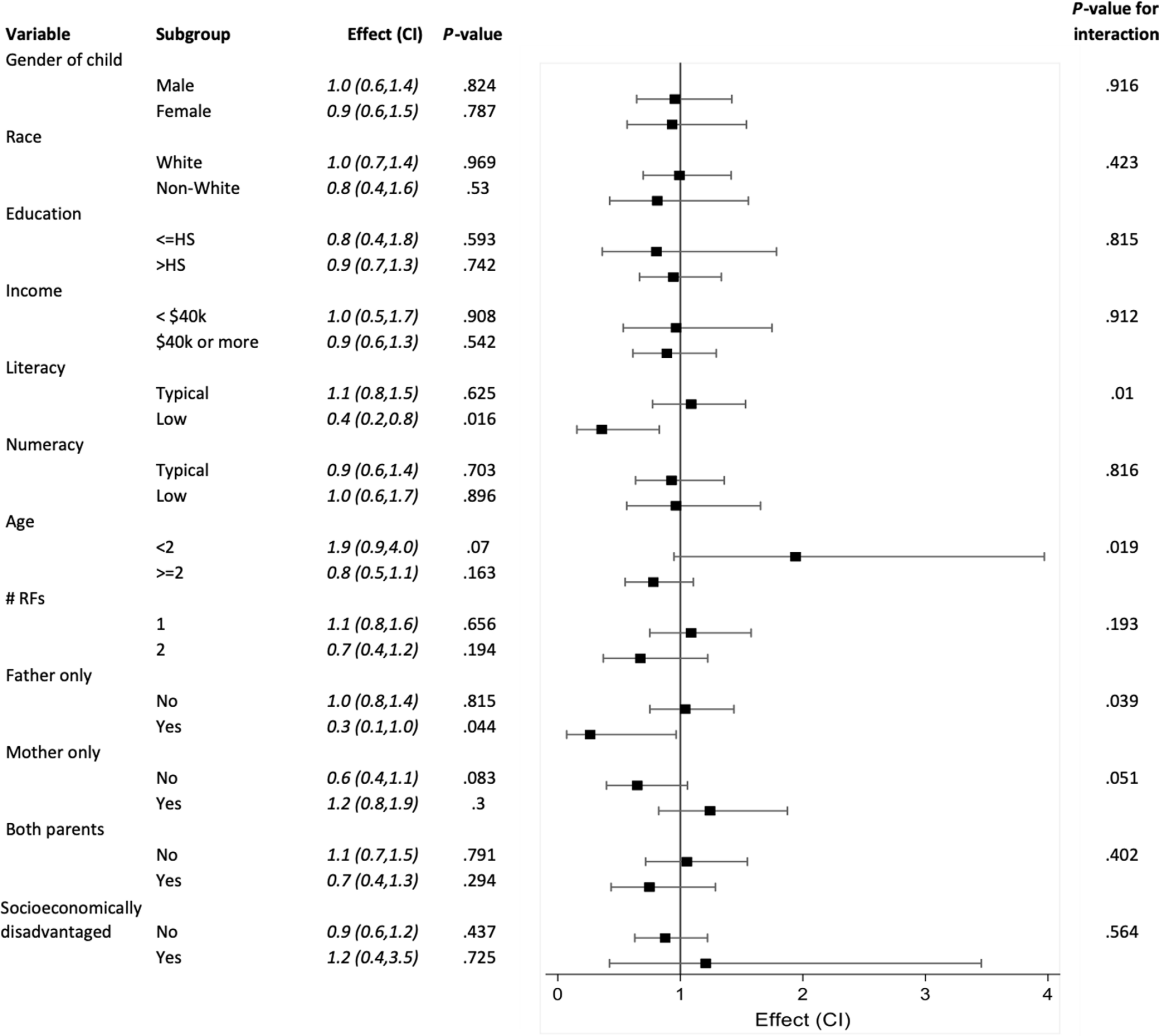
Cranial CT rate subgroup effects. Forest plot demonstrating the effect of the *Head CT Choice* decision aid on the rate of cranial computed tomography imaging obtained in subgroups according to patient and parent characteristics

Regarding diagnostic decisions, we identified significant interactions between whether a cranial CT was obtained and parent literacy, age of the child, and father only present during the encounter (Table 3). Children of parents with low health literacy had a lower odds of having a cranial CT performed compared to parents with high health literacy. On further analysis, 58 (24%) of non‐whites and 84 (12%) of whites had low health literacy (*P* < .001). If the child was less than 2 years of age, there was a higher odds of cranial CT compared to children 2‐18 years of age. Conversely, there was also a lower odds of cranial CT among encounters in which only the father was present compared to encounters with both parents and only the mother present (Figure 4).

**Table 3:** Differential effect of the decision aid on the emergency department (ED) cranial CT rate based on patient and parent sociodemographic characteristics

Characteristic	Decision Aid Effect **(**OR, 95% CI)	*P* value
Sex of child^a^		
Male	0.96 (0.64, 1.42)	.916
Female	0.93 (0.57, 1.54)	
Race		
White	0.99 (0.70, 1.41)	.423
Non‐white	0.81 (0.42, 1.56)	
Education		
≤HS	0.80 (0.36, 1.79)	.815
>HS	0.94 (0.67, 1.33)	
Income		
<$40k	0.97 (0.53, 1.75)	.912
≥$40k or more	0.89 (0.61, 1.29)	
Literacy		
Typical	1.09 (0.77, 1.53)	.010
Low	0.36 (0.16, 0.83)^b^	
Numeracy		
Typical	0.93 (0.63, 1.36)	.816
Low	0.96 (0.56, 1.65)	
Age^a^		
<2	1.94 (0.95, 3.97)	.019
≥2	0.78 (0.55, 1.11)	
Number of PECARN risk factors^a^		
1	1.09 (0.75, 1.58)	.193
2	0.67 (0.37, 1.22)	
Father only		
No	1.04 (0.75, 1.44)	.039
Yes	0.26 (0.07, 0.97)^b^	
Mother only		
No	0.65 (0.40, 1.06)	.051
Yes	1.24 (0.82, 1.87)	
Both parents		
No	1.05 (0.72, 1.55)	.402
Yes	0.75 (0.43, 1.29)	
Socioeconomically disadvantaged		
No	0.88 (0.63, 1.22)	.564
Yes	1.21 (0.42, 3.46)	

Abbreviations: CI, confidence interval; OR, odds ratio; PECARN, Pediatric Emergency Care Applied Research Network.

a Indicates a patient characteristic.

b Indicates a significant decision aid effect for the subgroup compared with its control (usual care) for the outcome. Reported if the *P* value for the overall interaction is significant.

## 4 DISCUSSION

In children with minor head trauma at intermediate risk of clinically important TBI according to the PECARN prediction rules (one or two intermediate PECARN risk factors),^10^ SDM facilitated by the *Head CT Choice* DA increased parent knowledge, decreased decisional conflict, increased parents’ trust in the clinician and facilitated greater clinician engagement of parents similarly across all parent/patient subgroups. Interestingly, in exploratory analyses the DA decreased decisional conflict to a relatively greater degree in non‐white parents and increased physician trust to a greater degree in socioeconomically disadvantaged parents. We also observed a lower odds of cranial CT imaging in children of parents with low health literacy and a higher odds of cranial CT in children younger than 2 years of age.

Use of the DA did not result in greater knowledge transfer in parents with higher numeracy as we had observed in our SDM trial of adults presenting to the ED with chest pain.^6^ Although the reasons for this observation are not clear, it is conceivable that paediatric emergency clinicians, on average, make greater efforts to educate parents in decision making compared to efforts made by emergency clinicians caring for adults. It is also possible parents of children with head trauma have a relatively greater degree of anxiety in the emergency setting compared to adults with non‐traumatic chest pain, impeding comprehension of numerical information during the encounter. The degree of difficulty of the knowledge test may also have differed between trials. Finally, the degree of reliance on numeracy may have differed between decision aids.

What might explain the greater decrease in decisional conflict among non‐white patients? In a cross‐sectional survey of 366 parents of children with life‐threatening illness, investigators found that black parents, compared to those that were white, had higher levels of decisional conflict.^19^ Findings from a qualitative study of African American patients with diabetes suggest that decisional conflict in black patients may be related to issues of physician mistrust and miscommunication.^7^ US history has unfortunately given African Americans reasons for mistrust that have not been sufficiently overcome by the modern health‐care state, as exemplified by the Tuskegee experiments that began in 1932 and went on for over 40 years.^20^ A telephone survey of adults who had a recent primary care visit also supports these findings. In this study, African American patients rated their visits as less participatory compared to whites. However, patients with race‐concordant relationships with their physicians rated their physicians as significantly more participatory.^21^ Engaging parents with higher levels of decisional conflict at baseline may result in a relatively greater improvement when engaged in SDM.

Why did use of the DA generate greater physician trust in socioeconomically disadvantaged parents? In our prior shared decision‐making trial in ED patients with chest pain, we observed greater physician trust in patients with low health literacy.^6^ Although the current trial was conducted in parents of children with minor head trauma, the DA was developed using the same methodology as in our prior trial in adults with chest pain, and both trials were conducted in the ED setting. For these reasons, we anticipated observing similar findings in the current trial. Although we did not observe the same finding in the current analysis, in the chest pain trial there was a greater proportion of non‐white patients, providing greater statistical power to detect this difference. From this perspective, the observation that use of the DA resulted in increased trust in socioeconomically disadvantaged parents is a similar finding observed in both trials.

We observed a lower odds of CT imaging in parents with low health literacy who were engaged in SDM. To the best of our knowledge, this finding has not been previously reported. However, findings from the PECARN head injury study of more than 42 000 children with minor head trauma from 25 North American EDs may provide some insight.^22^ In this study, children of black non‐Hispanic or Hispanic race/ethnicity had a lower odds of undergoing cranial CT imaging. In our trial, a greater proportion of non‐white parents had low health literacy, suggesting an association between non‐white race and low health literacy. Other investigators have also documented an association between race and health literacy. Shea and colleagues, in a prospective sample of 1610 primary care patients, documented lower Rapid Estimate of Adult Literacy in Medicine (REALM) scores in African Americans adults compared to Caucasians, even after stratifying by level of education.^23^

The odds of CT imaging was also higher in children younger than 2 years of age. However, there were no differences in knowledge, decisional conflict, physician trust or OPTION scores among parents of children younger than two or older than 2 years of age. This suggests that the higher rates of CT imaging in the younger age group may be related to greater parental anxiety and/or clinician uncertainty when caring for preverbal children who are unable to clearly express their symptoms.

### 4.1 Limitations and strengths of the study

The primary limitations of this study relate to issues of multiple testing and imprecision (lack of power) around estimates of subgroup effects. Given that a total of 55 comparisons were performed, one would expect 2‐3 (5% of 55) tests to be significant at the 5% level due to chance alone. To mitigate the risk of spurious findings related to multiple testing, we pre‐specified hypotheses based on prior observations in SDM trials.^3^, ^5^, ^6^ We also followed guideline recommendations for reporting subgroup analyses in clinical trials^18^ by distinguishing subgroup analyses of special interest in the methods, basing subgroup analyses on tests for interaction and cautiously interpreting subgroup differences. Our analyses often yielded imprecise results of potentially important subgroup effects. The limitation of imprecision is inherent in subgroup analyses of clinical trials, and, to the best of our knowledge, the current trial represents the largest cohort of parents enrolled in a SDM trial to date and has the potential to reveal important insights about subgroup effects of a DA in a paediatric population.

### 4.2 Implications for practice and future research

What are the implications of the findings of this subgroup analysis? It is possible that use of the *Head CT Choice* DA increased clinicians’ efforts to share information and engage parents in deliberations regarding whether imaging should be obtained for their child, and that this change in clinician behaviour mitigated parent distrust related to socioeconomic and racial disparities. For practicing clinicians, it is important to note that efforts to engage parents in SDM have potential to increase trust, particularly in socioeconomically disadvantaged parents, and that this trust can positively affect the therapeutic relationship. For researchers, the observations of lower decisional conflict in non‐white parents and greater physician trust in socioeconomically disadvantaged parents are exploratory. Additional research is needed to replicate these findings and to gain greater insight into how the SDM process alters the experiences and perceptions of care in socioeconomically disadvantaged populations.

## 5 CONCLUSIONS

Use of the *Head CT Choice* DA was similarly effective in all parent/patient dyads but did not increase knowledge more in parents with high numeracy or physician trust more in parents with low health literacy. In exploratory analyses, we found that decisional conflict was lower in non‐white parents, physician trust was greater in socioeconomically disadvantaged parents, and CT rates were lower in children whose parents had low health literacy. DAs may be particularly effective in potentially vulnerable parents.

## CONFLICTS OF INTERESTS

None of the authors have conflicts of interest that are directly relevant to the content of this article.

## AUTHOR CONTRIBUTIONS

RS was responsible for drafting the manuscript, and all authors were responsible for revising the manuscript critically for important intellectual content. EH was responsible for this study's conception. JH was responsible for data analysis. All authors were responsible for the acquisition and interpretation of data. All authors read and approved the final manuscript.

## Data Availability

The data that support the findings of this study are available on request from the corresponding author. The data are not publicly available due to privacy or ethical restrictions.
